# Neurometabolic Profile in Obese Patients: A Cerebral Multi-Voxel Magnetic Resonance Spectroscopy Study

**DOI:** 10.3390/medicina60111880

**Published:** 2024-11-16

**Authors:** Miloš Vuković, Igor Nosek, Johannes Slotboom, Milica Medić Stojanoska, Duško Kozić

**Affiliations:** 1 Faculty of Medicine, University in Novi Sad, 21000 Novi Sad, Serbia; igor.nosek@mf.uns.ac.rs (I.N.); milica.medicstojanoska@kcv.rs (M.M.S.); dusko.kozic@mf.uns.ac.rs (D.K.); 2 Institute for Diagnostic and Interventional Neuroradiology, University Hospital Bern and Inselspital, 3010 Bern, Switzerland; johannes.slotboom@gmail.com

**Keywords:** neurodegeneration, brain, neuroinflammation, metabolic syndrome, insulin, magnetic resonance spectroscopy

## Abstract

*Background and Objectives:* Obesity-related chronic inflammation may lead to neuroinflammation and neurodegeneration. This study aimed to evaluate the neurometabolic profile of obese patients using cerebral multivoxel magnetic resonance spectroscopy (mvMRS) and assess correlations between brain metabolites and obesity markers, including body mass index (BMI), waist circumference, waist-hip ratio, body fat percentage, and indicators of metabolic syndrome (e.g., triglycerides, HDL cholesterol, fasting blood glucose, insulin, and insulin resistance index (HOMA-IR)). *Materials and Methods:* This prospective study involved 100 participants, stratified into two groups: 50 obese individuals (BMI ≥ 30 kg/m^2^) and 50 controls (18.5 ≤ BMI < 25 kg/m^2^). Anthropometric measurements, body fat percentage, and biochemical markers were evaluated. All subjects underwent long- and short-echo mvMRS analysis of the frontal and parietal supracallosal subcortical and deep white matter, as well as the cingulate gyrus, analyzing NAA/Cr, Cho/Cr, and mI/Cr ratios, along with absolute concentrations of NAA and Cho. *Results:* Obese participants exhibited significantly decreased NAA/Cr and Cho/Cr ratios in the deep white matter of the right cerebral hemisphere (*p* < 0.001), while absolute concentrations of NAA and Cho did not differ significantly between groups (*p* > 0.05). NAA levels showed negative correlations with more reliable obesity parameters (waist circumference and waist-to-hip ratio) but not with BMI, particularly in the deep frontal white matter and dorsal anterior cingulate gyrus of the left cerebral hemisphere. Notably, insulin demonstrated a significant negative impact on NAA (ρ = −0.409 and ρ = −0.410; *p* < 0.01) and Cho levels (ρ = −0.403 and ρ = −0.392; *p* < 0.01) at these locations in obese individuals. *Conclusions:* Central obesity and hyperinsulinemia negatively affect specific brain regions associated with cognitive and emotional processing, while BMI is not a reliable parameter for assessing brain metabolism.

## 1. Introduction

Obesity presents a significant public health concern, with 300 million individuals classified as obese worldwide and over a billion people falling into the category of being overweight [1,2]. This condition is closely linked to a cluster of disorders known as metabolic syndrome [3], which is believed to be a common factor in various obesity-related illnesses due to its role in causing low-grade systemic inflammation that impacts multiple organs [4] and is connected to a heightened risk of dementia [5]. In cases of obesity, inflammatory reactions within the central nervous system, commonly known as neuroinflammation, have been observed in different areas such as the hypothalamus [6].

A study utilizing population-based MRI scans revealed that obesity and a high waist-to-hip ratio during middle age were linked to an increased likelihood of reduced brain volume and a decline in executive function a decade later. Furthermore, the metabolic obesity profile, marked by increased body fat, visceral adiposity, and systemic inflammation, was linked to a widespread reduction in gray matter volume [7]. Additionally, diabetes was associated with a faster increase in temporal horn volume, which serves as a surrogate marker for accelerated hippocampal atrophy [8]. Previous studies have indicated that greater weight and central obesity are correlated with diminished brain volume [9,10,11,12]. Furthermore, a higher body mass index (BMI) and waist circumference have been found to be significantly linked to a thinner cerebral cortex [13].

It is well known that magnetic resonance spectroscopy (MRS) brings additional information to conventional neuroimaging in clinical practice. This method can detect the neurometabolic profile of the lesion and its spatial heterogeneity [14]. Multivoxel MRS (mvMRS) is a sophisticated diagnostic modality in neuroscience that can reveal marked metabolic abnormalities in examinees with no morphological changes on conventional MR imaging, even in mutation carriers of genetic disorders [15]. Ostojic et al. found marked differences among the head, body, and tail of the hippocampus in healthy persons, compatible with different histologic characteristics of the aforementioned segments [16]. Multivoxel MRS can detect significant disturbances in neurometabolic ratios in various disorders, including traumatic brain injury, HIV, and other diseases, although morphologic changes were not evident on routine scanning [17].

It is imperative to comprehend the neurometabolic state of individuals who are obese but show no neurological symptoms. By utilizing MRS, we can non-invasively determine the biochemical profile of brain tissue, which provides insights into both its structural and functional abnormalities. This technique relies on measuring the absolute and relative concentrations of specific metabolites, namely N-acetylaspartate (NAA), choline-containing molecules (Cho), creatine (Cr), myoinositol (mI), glutamate, lactate, and lipid components [18,19].

Brain atrophy and reduced levels of NAA, particularly in the temporal lobes and hippocampus, are considered risk factors for cognitive decline and dementia in older individuals. N-acetylaspartate serves as an established indicator of neuronal viability, only present in mature neurons and their extensions. A decrease in its concentration signifies neuronal loss and dendritic and axonal atrophy. On the other hand, choline-containing compounds primarily play a role in the breakdown and synthesis of cell membranes. Metabolites containing Cr are involved in cellular bioenergetics, while mI is regarded as a marker for the number of glial cells (indicative of neuroinflammation) and an osmoregulator [1].

We analyzed and compared individuals with obesity to those with a normal BMI. We examined the absolute concentrations of NAA and Cho in relation to various anthropometric parameters such as BMI, waist circumference, waist-hip ratio, and body fat percentage in both groups. Additionally, we assessed these concentrations in relation to biochemical markers of metabolic syndrome in the obese participants, including triglycerides, HDL cholesterol, fasting blood glucose and insulin levels, as well as the insulin resistance index (HOMA-IR).

## 2. Materials and Methods

### 2.1. Participants in the Study

A total of 100 subjects were included in the study, divided into two groups:

1. The first group consisted of 50 obese persons, with a BMI of ≥30 kg/m^2^ (25 men and women each), average age 43.32 years (22–62).
2. The second group consisted of 50 control subjects with BMI ≥ 18.5 and <25 kg/m^2^ (25 men and women each), average age 43.22 years (23–62).

This study was a prospective cohort study that included participants who did not have any neurological deficits based on the Montreal Cognitive Assessment (MoCA) test [20]. The subjects in both groups were sex- and age-matched, aged between 20 and 65. Exclusion criteria for both groups included acute or chronic neurological disorders, mood disorders, anxiety [21,22], white matter lesions, and the use of medications affecting lipid levels, blood glucose, and insulin (glucocorticoids, statins, fibrates, oral hypoglycemics, and GLP-1 receptor agonists), as well as conditions such as Cushing’s syndrome, acromegaly, and uncontrolled hypothyroidism.

Contraindications for an MRI exam included both absolute and relative factors. Absolute contraindications were implanted devices such as pacemakers (unless MRI-compatible), neurostimulators, cochlear implants, and metallic objects near the eyes. Relative contraindications included claustrophobia, first-trimester pregnancy, obesity beyond machine limits, and difficulty remaining still.

The study was approved by the institutional ethical review board, and all participants provided informed consent before joining the study.

### 2.2. Anthropometric Measurements

The height and weight of all participants in the study were measured, and the BMI was calculated (formula: weight/height^2^ in kg/m^2^). Additionally, waist circumference (WC) was measured, as well as the waist-hip ratio. Waist circumference was measured halfway between the lower rib and the iliac crest along the midaxillary line, while hip circumference was measured at the widest point over the greater trochanters. Furthermore, body fat percentage was measured by using a bioelectrical impedance body composition analyzer, TBF-300, Tanita, Tokyo, Japan.

### 2.3. Laboratory Tests and Other Measurements

All obese participants in the study underwent an analysis of biochemical indicators of metabolic syndrome (triglyceride, HDL cholesterol, blood glucose, and insulin with calculation of the HOMA-IR index). For insulin measurement, we used the chemiluminescent microparticle immunoassay (CMIA) method. Blood samples were taken from the cubital vein 12 h after fasting. The HOMA-IR index was calculated according to the formula HOMA-IR = (fasting blood glucose × fasting insulinemia)/22.5.

### 2.4. Neuroimaging

All subjects underwent conventional MR imaging with additional mvMRS on a 1.5 Tesla magnetic field device (Magnetom Avanto, Siemens, Erlangen, Germany) using a head coil. Conventional MR imaging consisted of T1W sagittal spin echo tomograms, T2W turbo spin echo transverse tomograms, FLAIR transverse tomograms, diffusion imaging (DWI), coronal T2W turbo spin echo tomograms, and 3D T1W MPR sagittal tomograms. Conventional imaging was used to exclude possible focal or diffuse white and gray matter lesions and to correctly position the region of interest for mvMRS (voxel grid).

**Figure 1:**
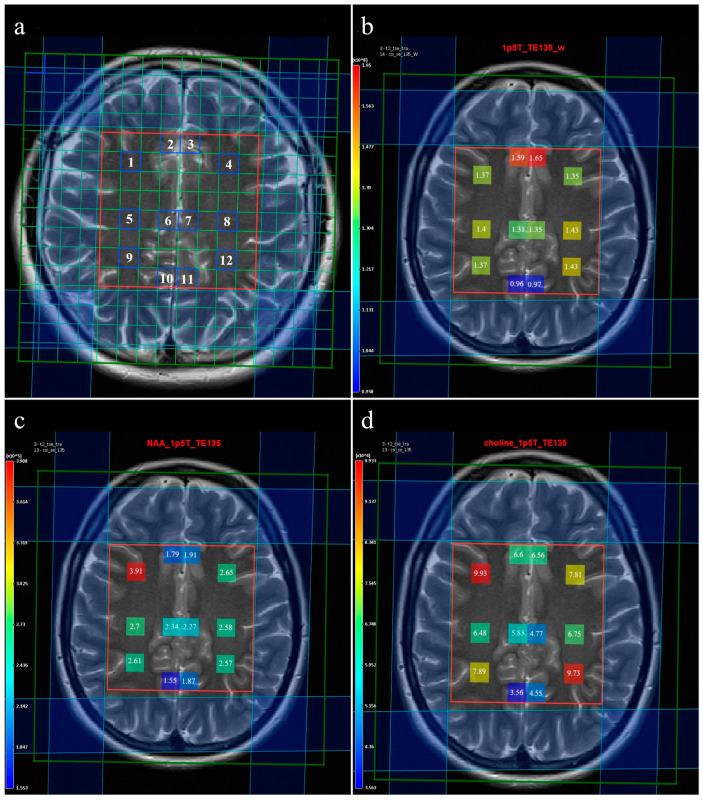
Processing of absolute metabolite concentrations of long-echo mvMRS: (**a**) numbered voxel of interest (VOI); (**b**) intensity of water at VOI; (**c**) intensity of NAA; (**d**) intensity of Cho.

Multivoxel MR spectroscopy of the supracallosal white and gray matter was performed in all subjects included in the study. Proton MR spectroscopy in the form of the Point RESolved Spectroscopy method (PRESS) was used to obtain data through spectroscopy, with TR/TE for long echo 1690/135 ms and for short echo 1690/30 ms. Section dimensions on CSI spectroscopic imaging were determined by the following parameters: FOV size was 160 mm × 160 mm × 160 mm, the volume of interest (VOI) was 80 mm × 80 mm × 80 mm, and section thickness was 10 mm. The voxel grid was placed directly above the corpus callosum in order to determine the spectra from the subcortical and deep white matter of the centrum semiovale of the frontal and parietal lobes, while the gray matter spectra were determined from the parafalx area of the anterior and posterior cingulate gyrus. The number of phase encoding steps was 16 in all directions (right-left, forward-backward, and up-down). The interpolation resolution was 16 in all directions, obtaining a VOI of 10 mm × 10 mm × 10 mm. To achieve the homogeneity of the magnetic field, automatic, volume-selective shimming was used. Non-water-suppressed CSI data were obtained with the same geometric parameters (1 average) to provide an internal water reference for the absolute quantification of metabolites. The amplitude of the water signal for each processed voxel was assessed from the scan without water suppression and used as an internal reference to calculate the absolute concentration of the metabolites. The SpectrImQMRS program, a tool for the combined analysis of MR spectroscopy and anatomy, was used to calculate the absolute concentrations of the investigated metabolites [23]. To process the spectra of metabolites, pre-processing was carried out, and the obtained values were expressed through the metabolite intensity or the area under the curve (Area) (Figure 1), and these data were further used to quantify the absolute values of the metabolites. A similar pre-processing procedure was also applied to the non-water-suppressed CSI spectra.

**Figure 2:**
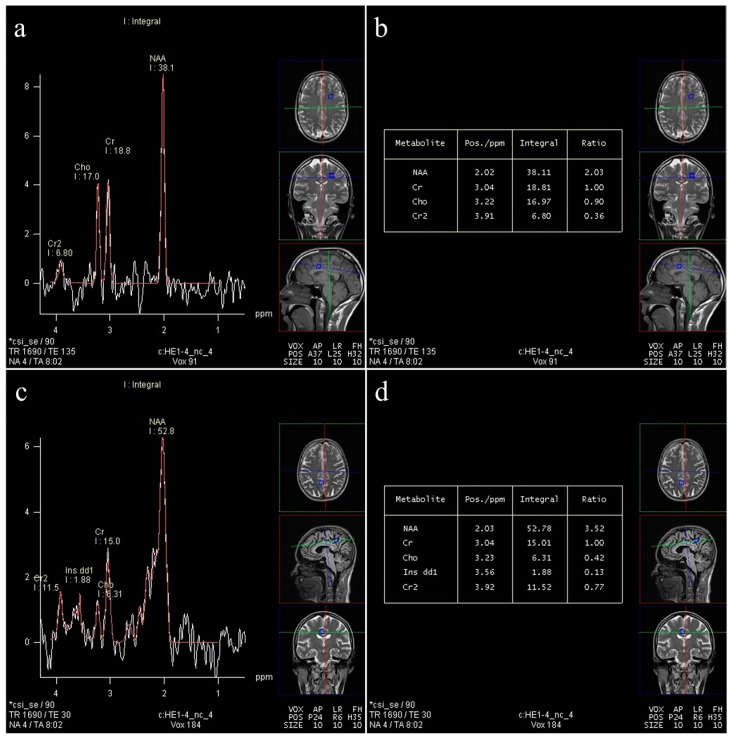
Normal spectrum and ratios in a control subject: (**a**,**b**) long-echo MRS in the left subcortical FWM (V4); (**c**,**d**) short-echo MRS of the right PCG (V10).

**Figure 3:**
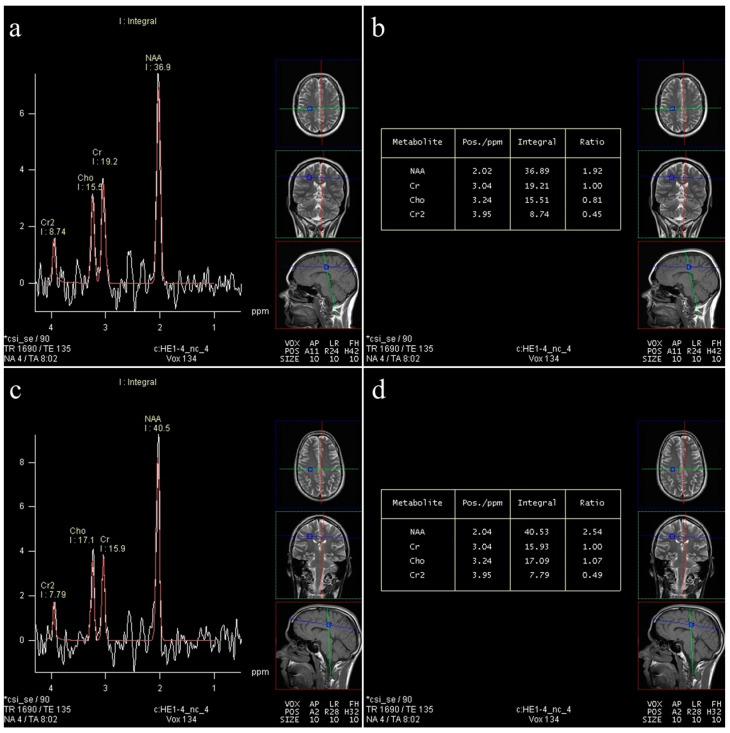
Long-echo mvMRS in the region of the right deep FWM (V5): spectrum (**a**) in an obese subject, showing a slight decrease in the NAA/Cr (1.92) and Cho/Cr ratio (0.81) (**b**); spectrum (**c**) in a control subject, showing a normal ratio of NAA/Cr (2.54) and Cho/Cr (1.07) (**d**).

**Figure 4:**
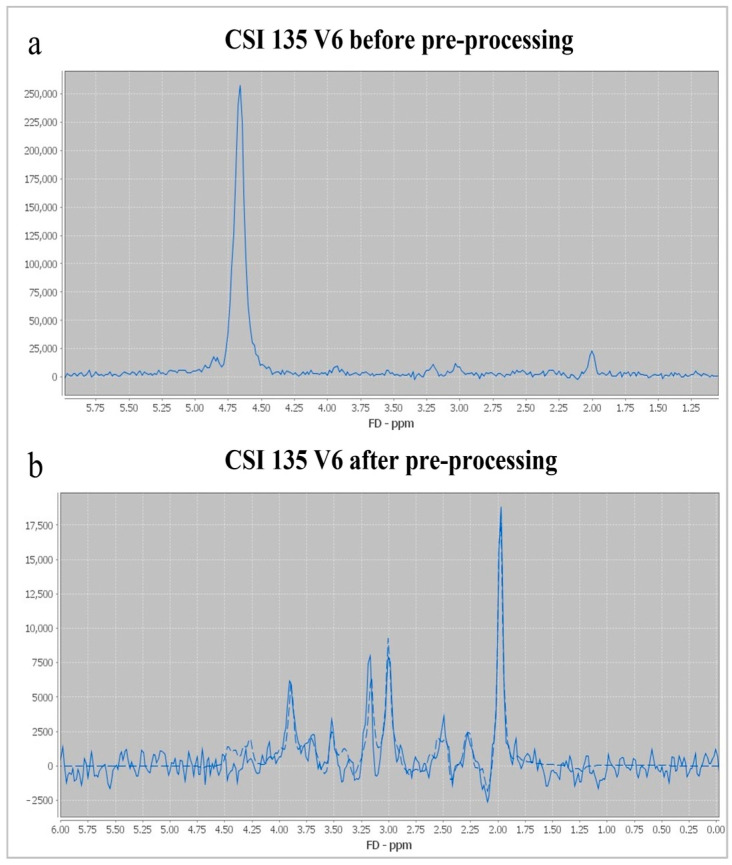
Determination of metabolite intensity through spectrImQMRS: (**a**)—spectrum from the posterior anterior cingulate gyrus (ACG) of the right cerebral hemisphere (V6) before pre-processing; (**b**)—the same spectrum after pre-processing and spectral fitting.

**Figure 5:**
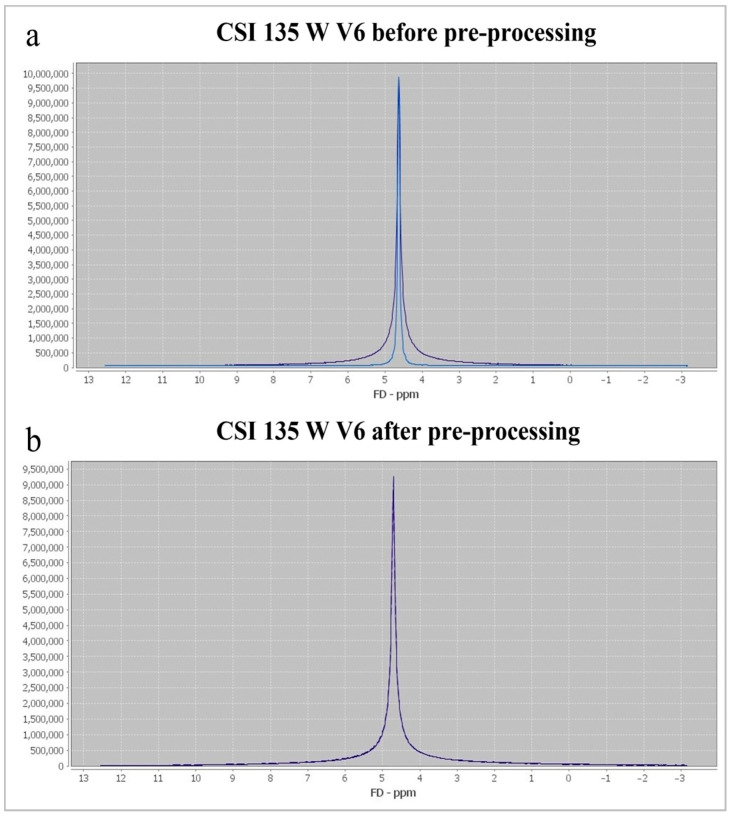
Determination of water intensity through spectrImQMRS: (**a**)—spectrum from the posterior anterior cingulate gyrus (ACG) of the right cerebral hemisphere (V6) without water suppression before pre-processing; (**b**)—the same spectrum after pre-processing and spectral fitting.

In total, about 4800 spectra were analyzed. We calculated the ratios of metabolites NAA/Cr, Cho/Cr, and mI/Cr from the Leonardo workstation (Figure 2 and Figure 3) and absolute concentrations of NAA at 2.0 parts per million (ppm) and Cho at 3.2 (ppm) using spectral fitting with the appropriate basis set model in the SpectrImQMRS program (Figure 4 and Figure 5) [24]. Monoexponential T1 and T2 relaxation was assumed, and published values of T1 and T2 relaxation times of water and corresponding metabolites measured at 1.5 T in gray and white matter of healthy volunteers were used for relaxation corrections [25]. The obtained absolute concentrations were expressed in mmol/L.

We analyzed spectra from 12 individual voxels (Figure 1a), namely: 1. subcortical frontal white matter (FWM) of the right cerebral hemisphere; 2. anterior cingulate gyrus (ACG) of the right cerebral hemisphere; 3. ACG of the left cerebral hemisphere; 4. subcortical FWM of the left cerebral hemisphere; 5. deep FWM of the right cerebral hemisphere; 6. the posterior ACG of the right cerebral hemisphere; 7. the posterior ACG of the left cerebral hemisphere; 8. deep FWM of the left cerebral hemisphere; 9. subcortical parietal white matter (PWM) of the right cerebral hemisphere; 10. posterior cingulate gyrus (PCG) of the right cerebral hemisphere; 11. PCG of the left cerebral hemisphere; 12. subcortical PWM of the left cerebral hemisphere.

### 2.5. Statistical Analysis

IBM SPSS software version 27.0 (Chicago, IL, USA) was used for statistical data processing. Since all subjects were imaged on the same MR machine, potential scanner-dependent differences between patients did not exist. Descriptive statistics were reported as the mean and standard deviation for variables that exhibited a normal distribution, while for those that did not conform to normality, the statistics were presented as the median and interquartile range. Determining the difference in neurobiochemical profile within the brain parenchyma between obese patients and controls was performed with the t-test or the Mann-Whitney U test depending on the normality of the distribution of the test variables, which was examined with the Kolmogorov Smirnov test. A value of 95% with a statistical significance level of *p* < 0.05 was taken as the confidence interval.

The examination of the relationship between the anthropometric parameters of all subjects, as well as the biochemical parameters of the metabolic syndrome of obese patients with the parameters in the neurobiochemical profile was analyzed with the Spearman correlation coefficient (ρ), because all anthropometric and biochemical parameters have abnormal distribution [26]. The correlation between age and parameters in the neurobiochemical profile of the participants was also examined using the Spearman correlation coefficient.

## 3. Results

### 3.1. Demographic, Anthropometric, and Biochemical Data

This prospective cohort study was conducted on 100 participants: 50 obese people with a BMI over 30 kg/m^2^ and 50 people with a normal BMI, between 18.5 and 24.9 kg/m^2^. An equal number of men and women were recruited in both groups (25 each), matching the participants for age and sex to exclude their influence on examined parameters (Table 1). The level of education can potentially influence the results of the examined parameters, so we recruited subjects with approximately similar educational levels between the groups (χ^2^ = 1.412, df = 1, *p* = 0.235).

The anthropometric measurements of all participants in the study are listed in Table 1, while Table 2 presents the biochemical indicators of metabolic syndrome for the obese participants.

### 3.2. Correlation Between Age and Neurometabolites

#### 3.2.1. Relative Concentrations

Although the obese and control groups are homogeneous in terms of age, an examination of the association of metabolites obtained by mvMRS long and short echo with age was performed, considering that literature data indicate that the level of certain metabolites changes with age (primarily NAA and Cho).

The results show that NAA/Cr values decrease with age in 6 out of 12 examined voxels, both for long-echo mvMRS and short-echo mvMRS, with a smaller deviation in localizations. The degree of correlation in statistically significant voxels was weak (ρ < 0.3), except for V9 (right subcortical PWM) on the short echo, where it was moderate (ρ = 0.347, *p* < 0.001).

Regarding the Cho/Cr ratio at almost all observed locations, no statistically significant association with age was found, with the exception of V11 (left PCG) on the short echo, where it can be seen that the level of Cho/Cr increases with age and that this correlation is of moderate intensity (ρ = 0.327, *p* < 0.001).

#### 3.2.2. Absolute Concentrations

The correlation of absolute NAA concentrations with age shows a statistically significant negative association at all voxels of interest, which confirms that NAA values decrease with age, independently of Cr, and that in half of the locations there is a strong correlation (ρ > 0.4).

Analyzing the correlation between age and absolute concentrations of Cho, a statistically significant negative correlation is observed in only three voxels: at voxels V4 and V11, weak correlation (ρ < 0.3), while at the V2 location it is moderate (0.3 < ρ < 0.3.99).

### 3.3. Comparison of Relative Concentrations of Metabolites Between Groups on Long-Echo MRS

#### 3.3.1. NAA/Cr

The results in Table 3 show that there is no difference in the values of the NAA/Cr ratio between the groups in almost all examined locations, with the exception of V5 (right deep FWM), where a statistically significantly lower value of NAA/Cr is observed in obese subjects (*p* < 0.001) (Figure 3a,b) in comparison to controls (Figure 3c,d).

#### 3.3.2. Cho/Cr

The results in Table 4 show the comparison of Cho/Cr ratio between the groups with no statistically significant difference in almost all examined locations with the exception of V5 (right deep FWM), where a statistically significantly lower value of Cho/Cr is observed in obese subjects (*p* = 0.009, *p* < 0.01) (Figure 3a,b).

### 3.4. Comparison of Absolute Concentrations of NAA and Cho Between Groups on Long-Echo MRS

Based on the obtained results, there is no statistically significant difference in absolute concentrations of NAA and Cho between obese subjects and controls at any of the studied locations of interest.

### 3.5. Comparison of Relative Concentrations of Metabolites Between Groups on Short-Echo MRS

Based on the obtained results, we did not find any difference in the values of the NAA/Cr and mI/Cr ratios between obese subjects and controls in all the examined locations. As for Cho/Cr, the absence of a statistically significant difference is observed in almost all examined locations except for V6 (right posterior ACG), where a statistically significantly lower value of Cho/Cr is observed in obese subjects (*p* = 0.037, *p* < 0.05).

### 3.6. Correlation of Absolute Concentration of NAA and Cho with Anthropometric Parameters and Body Fat Percentage in Subjects of Both Groups on Long-Echo MRS

There is a weak negative correlation with waist circumference at V7 and V8 (*p* = 0.021), with waist-hip ratio at V6 (*p* = 0.015), V7 and V8 (*p* = 0.017), as well as with body fat percentage at V4 (*p* = 0.023). The obtained results show us that the parameters that more clearly reflect obesity (waist circumference and waist-hip ratio) have more influence on NAA values than BMI (Table 5).

As for Cho, the results show there is a weak negative correlation only with the percentage of body fat at position V4 (left subcortical FWM) (*p* = 0.016).

### 3.7. Correlation of Absolute Concentrations of NAA and Cho with Biochemical Indicators of Metabolic Syndrome in Obese People on Long-Echo MRS

#### 3.7.1. NAA

The results shown in Table 6 demonstrate that there is a strong negative correlation of the NAA concentrations with the insulin level at the V7 (*p* = 0.006) and V8 (*p* = 0.007) positions, as well as the HOMR-IR index level at the same locations (*p* = 0.005). A slightly contradictory result is the positive correlation of NAA values with insulin at the V4 position, but it is borderline weak to moderate intensity (*p* = 0.047).

#### 3.7.2. Cho

The results shown in Table 7 demonstrate that there is a negative correlation of a moderate degree of Cho value with the level of insulin at the position of V7 (left posterior ACG) (*p* = 0.009) and a strong degree at V8 (left deep FWM) (*p* = 0.007), as well as with the level of the HOMR-IR index at the same locations of a moderate degree (*p* = 0.023 and *p* = 0.019). From the previously presented results, it can be observed that NAA and Cho values are negatively correlated with insulin and the HOMA-IR index at V7 and V8, which indicates their influence on degenerative processes at those locations. The result, which is a little contradictory, is a positive correlation of a moderate intensity of Cho with triglycerides at the V5 position (*p* = 0.036).

## 4. Discussion

There are no studies in the literature that have examined the association of biochemical indicators of metabolic syndrome (triglyceride, HDL cholesterol, blood glucose, and insulin level, as well as the insulin resistance index (HOMA-IR)) in obese patients with cerebral metabolite concentrations obtained by the mvMRS. There are also no literature data that compare the concentrations of brain metabolites with the percentage of body fat.

There are various mechanisms by which obesity can impact brain health [27,28], particularly regarding the structure of gray matter [29]. First, obesity is associated with hypertension, diabetes, and hyperlipidemia, which are known causes of poor brain health. Second, obesity-associated low-grade chronic inflammation may induce neuroinflammation [30,31]. Different structures of the central nervous system could be affected, including the cerebral cortex, amygdala, cerebellum, and hypothalamus [32,33]. Brain involvement is most likely related to the disturbed blood-brain barrier, with consequent entry of pro-inflammatory cytokines to the brain parenchyma [34] and activation and proliferation of microglia and astrocytes. Microglial proliferation has been previously reported in neurodegenerative diseases and traumatic brain injury [35]. The obesity-related microglial proliferation induces synaptic remodeling, suppressing neurogenesis, leading to cognitive decline [36].

Analyzing the difference in the level of metabolites between obese subjects and controls, we found that only right deep FWM on long-echo MRS has statistically significantly lower values of NAA/Cr and Cho/Cr. Looking at this finding, the obtained results could indicate initial neurodegenerative changes, but a comparison of the absolute values of NAA and Cho between the examined groups did not show the presence of a statistically significant difference. Comparison of mI/Cr ratio between the obese subjects and controls did not find a statistically significant difference. Myoinositol is found mainly in astrocytes, and its high concentrations are interpreted as a reflection of glial proliferation or an increase in the size of glial cells; therefore, it is considered a marker of neuroinflammation [37], which indicates that in our study, there is an absence of a neuroinflammation state between the groups. In another study, elevated concentrations of myoinositol were reported in metabolic syndrome as well as in type 2 diabetes [38].

First of all, it should be noted that there is a negative correlation of NAA values with age, which is in agreement with the results of other studies. This data should be taken into account when analyzing other correlations in order to get a real picture of the relationship between the investigated parameters.

One of the main studies that compared BMI with metabolite values obtained on single-voxel MRS found that higher BMI was significantly associated with lower NAA concentration in frontal, parietal, and temporal white matter, with lower NAA concentration in frontal gray matter, and with lower concentration of choline in the frontal white matter. This study found no association of BMI with regional concentrations of creatine or myoinositol [1]. In contrast to the results of the mentioned study, in our research we did not find that the BMI of participants correlated with the absolute concentrations of NAA in any of the investigated locations. On the other hand, looking at other anthropometric parameters such as waist circumference and waist-hip ratio, we obtained a negative correlation with the level of NAA at positions V7 (left posterior ACG) and V8 (left deep FWM). However, these results were not followed by a correlation of Cho levels. From the above, we can conclude that BMI is not a true parameter that reflects real obesity, that waist circumference and waist-hip ratio better reflect central obesity, and that they correlate better with NAA values in the described locations. Additionally, the waist-hip ratio negatively correlates with NAA values at position V6 (right posterior ACG), and the percentage of body fat correlates negatively with NAA only at position V4 (left subcortical FWM). From these results, we see that the percentage of body fat is an unreliable parameter that reflects obesity, similar to BMI, and perhaps for this reason we did not find a correlation with the values of NAA and Cho.

Analysis of the correlation of biochemical indicators of metabolic syndrome (triglycerides, HDL cholesterol, blood glucose, and insulin with the HOMA-IR index) with absolute concentrations of NAA and Cho in obese individuals showed interesting results. Two locations in the brain parenchyma showed a strong negative correlation between NAA and Cho values with insulin levels and the HOMA-IR index, namely left posterior ACG and left deep FWM. If we look at the correlations with the anthropometric parameters, we see that significant correlations are in the same locations. Changes in NAA levels can be caused by an insulin disorder, i.e., the presence of insulin resistance or hyperinsulinemia, which often exists in obese people [39]. This insulin disorder leads to impaired insulin transport in the brain, which in turn leads to impaired glucose utilization in the brain, which can be associated with lower NAA values [40]. Previous studies have shown low concentrations of NAA in patients with glucose intolerance [41] or diabetes [19,41]. A study examining metabolite concentrations in metabolic syndrome found a decreased ratio of NAA/Cr and an increased ratio of Cho/Cr in the white matter of the frontal lobe in patients with metabolic syndrome compared to patients without it, with the changes being more pronounced in obese patients [42]. In adolescents with metabolic syndrome, the levels of NAA, choline, and myoinositol were significantly reduced in both hippocampi, especially in the right hippocampus [43]. Choline-containing compounds are primarily involved in the breakdown and synthesis of cell membranes, and their concentrations are elevated in type 2 diabetes [44].

One study that examined the reversibility of brain metabolite changes after intragastric balloon insertion found that in a combined group of obese people with and without diabetes, changes in the NAA/Cr ratio during the first 3 months after intragastric balloon insertion were inversely proportional to changes in body weight and BMI. Furthermore, patients with type 2 diabetes had an elevated mI/Cr ratio, which was found to normalize only 3 months after intragastric balloon insertion. This change was accompanied by a decrease in the Cho/Cr ratio. N-acetylaspartate concentrations, but not myoinositol, have been shown to be affected by diet [45,46].

Creatine (Cr) has long been considered a constant in the brain, and metabolite values are expressed through the relationship with Cr. In our study, the analysis of absolute concentrations shows that the only explanation for the mentioned difference in the NAA/Cr and Cho/Cr ratios would be an increase in Cr in obese people in the right deep FWM, which leads to lower metabolic ratios. The possible explanation for this phenomenon could be the generally increased energy and caloric intake in the obese and the consequent higher amount of glucose, i.e., energy in the brain. As Cr is considered to have a role in the storage and transfer of energy in metabolically active tissues, such as the brain, muscles, and heart, it is possible that in obese people there is an excess of Cr in the right deep FWM, i.e., the centrum semiovale, where white matter tracts transit, of which the most important is the corticospinal tract.

One of the important limitations of the study is the use of a 1.5 T MRI scanner, instead of 3 T, which may limit the sensitivity of our study due to the relatively low signal-to-noise ratio (SNR) of MRS at this field strength. This reduced SNR can impair the detection of metabolites and other subtle changes, potentially impacting the accuracy and reliability of the results. Furthermore, an examination of the level of intelligence for the participants in the study was not carried out, which could eventually lead to a difference between the metabolites in the brain, but care was taken to ensure that the obese and control groups did not differ in terms of their level of education. Potential confounding factors, such as hypertension, lifestyle habits, dietary patterns, and levels of physical activity, should be considered too, as they may influence the brain metabolism. Additionally, one of the limitations of the study is the absence of more detailed cognitive and behavioral testing, which would allow for a more comprehensive interpretation of the results, given that obesity has been shown to affect deficits in cognitive control [47].

## 5. Conclusions

This research is one of the rare prospective studies to observe the impact of obesity on several different locations in the brain, and we concluded that specific changes are noticed in the right deep FWM. However, when analyzing absolute concentrations of neurometabolites, it seems that they are not a reflection of neurodegenerative processes (lower NAA/Cr and Cho/Cr). Instead, there is most likely an increased energy metabolism in the obese primarily due to an increase in Cr in the white matter pathways. Central obesity (a higher waist circumference and waist-to-hip ratio) and hyperinsulinemia have negative effects in the context of neurodegeneration on the deep left FWM and the left posterior ACG, which is responsible for cognitive and emotional processing and which can eventually lead to Alzheimer’s disease, while BMI is not a reliable parameter for assessing brain metabolism.

Future research should focus on clarifying the relationship between neurometabolic changes in obesity and a broader range of cognitive and behavioral assessments. Additionally, studies should expand neurometabolic assessments to other brain regions, particularly the hippocampus, and explore the connection between these neurometabolic alterations and potential microstructural and volumetric brain changes in individuals with obesity.

## Figures and Tables

**Table 1:** Age and anthropometric characteristics of all participants in the study.

	Groups	Mean (SD)	Median (IQR)	*p*
Age	Obese	43.32 (11.01)	N/A *	0.950
	Controls	43.22 (11.09)	N/A	
BMI	Obese	36.19 (4.33)	N/A	<0.001
	Controls	22.23 (1.39)	N/A	
Waist circumference (cm)	Obese	115.78 (10.44)	N/A	<0.001
	Controls	N/A	79.00 (8.50)	
Waist-to-hip ratio	Obese	0.94 (0.07)	N/A	<0.001
	Controls	0.85 (0.05)	N/A	
Body fat (%)	Obese	N/A	41.10 (13.02)	<0.001
	Controls	24.81 (6.77)	N/A	

* N/A—not applicable

**Table 2:** Biochemical indicators of metabolic syndrome in obese participants.

	Median	Interquartile Range (IQR)
Triglycerides (mmol/L)	1.37	0.90
HDL cholesterol (mmol/L)	0.99	0.33
Glucose (mmol/L)	4.95	0.88
Insulin (µlU/mL)	10.30	4.70
HOMA-IR	2.20	1.25

**Table 3:** Differences in NAA/Cr ratios obtained by the long-echo mvMRS between the groups.

	Groups	Mean (SD)	Median (IQR)	*p*
NAA/Cr 1	Obese	1.967 (0.431)	N/A	0.972
	Controls	1.970 (0.427)	N/A	
NAA/Cr 2	Obese	1.611 (0.364)	N/A	0.052
	Controls	1.467 (0.367)	N/A	
NAA/Cr 3	Obese	1.532 (0.287)	N/A	0.936
	Controls	1.525 (0.466)	N/A	
NAA/Cr 4	Obese	1.949 (0.378)	N/A	0.127
	Controls	2.064 (0.370)	N/A	
NAA/Cr 5	Obese	2.219 (0.447)	N/A	<0.001
	Controls	2.586 (0.423)	N/A	
NAA/Cr 6	Obese	N/A	1.480 (0.44)	0.250
	Controls	1.609 (0.301)	N/A	
NAA/Cr 7	Obese	1.506 (0.259)	N/A	0.282
	Controls	1.560 (0.246)	N/A	
NAA/Cr 8	Obese	2.184 (0.452)	N/A	0.094
	Controls	2.324 (0.371)	N/A	
NAA/Cr 9	Obese	2.044 (0.306)	N/A	0.114
	Controls	1.949 (0.287)	N/A	
NAA/Cr 10	Obese	1.624 (0.307)	N/A	0.575
	Controls	1.663 (0.375)	N/A	
NAA/Cr 11	Obese	N/A	1.570 (0.33)	0.236
	Controls	1.651 (0.328)	N/A	
NAA/Cr 12	Obese	1.827 (0.366)	N/A	0.535
	Controls	N/A	1.815 (0.55)	

**Table 4:** Differences in Cho/Cr ratios obtained by the long-echo mvMRS between the groups.

	Groups	Mean (SD)	Median (IQR)	*p*
Cho/Cr 1	Obese	N/A	1.210 (0.50)	0.331
	Controls	1.206 (0.239)	N/A	
Cho/Cr 2	Obese	N/A	1.120 (0.32)	0.155
	Controls	N/A	1.030 (0.43)	
Cho/Cr 3	Obese	1.094 (0.234)	N/A	0.942
	Controls	N/A	1.100 (0.31)	
Cho/Cr 4	Obese	1.254 (0.297)	N/A	0.406
	Controls	1.303 (0.294)	N/A	
Cho/Cr 5	Obese	N/A	1.105 (0.30)	0.009
	Controls	1.295 (0.281)	N/A	
Cho/Cr 6	Obese	N/A	1.045 (0.29)	0.735
	Controls	1.034 (0.212)	N/A	
Cho/Cr 7	Obese	1.028 (0.189)	N/A	0.424
	Controls	0.996 (0.209)	N/A	
Cho/Cr 8	Obese	1.260 (0.304)	N/A	0.817
	Controls	1.247 (0.243)	N/A	
Cho/Cr 9	Obese	1.077 (0.232)	N/A	0.169
	Controls	1.015 (0.213)	N/A	
Cho/Cr 10	Obese	N/A	0.750 (0.22)	0.051
	Controls	0.857 (0.238)	N/A	
Cho/Cr 11	Obese	0.780 (0.271)	N/A	0.471
	Controls	0.816 (0.218)	N/A	
Cho/Cr 12	Obese	0.983 (0.231)	N/A	0.294
	Controls	1.036 (0.272)	N/A	

**Table 5:** Correlation of absolute concentrations of NAA obtained by long-echo mvMRS with anthropometric parameters and body fat percentage.

Long-Echo		BMI	Waist Circumference	Waist-to-Hip Ratio	Body Fat (%)
NAA 1	ρ	0.079	0.053	−0.051	0.010
	*p*	0.450	0.617	0.624	0.921
	N	94	94	94	94
NAA 2	ρ	0.050	0.037	−0.023	0.033
	*p*	0.634	0.727	0.826	0.751
	N	94	94	94	94
NAA 3	ρ	0.023	0.086	0.125	0.086
	*p*	0.825	0.415	0.232	0.411
	N	94	94	94	94
NAA 4	ρ	−0.072	−0.029	0.071	−0.235 *
	*p*	0.495	0.783	0.497	0.023
	N	94	94	94	94
NAA 5	ρ	−0.085	−0.086	0.056	−0.188
	*p*	0.419	0.413	0.595	0.071
	N	94	94	94	94
NAA 6	ρ	−0.102	−0.172	−0.251 *	−0.044
	*p*	0.330	0.099	0.015	0.676
	N	94	94	94	94
NAA 7	ρ	−0.187	−0.239 *	−0.246 *	−0.089
	*p*	0.073	0.021	0.017	0.399
	N	94	94	94	94
NAA 8	ρ	−0.188	−0.240 *	−0.248 *	−0.088
	*p*	0.072	0.021	0.017	0.399
	N	94	94	94	94
NAA 9	ρ	−0.029	−0.065	−0.085	−0.084
	*p*	0.786	0.535	0.419	0.422
	N	94	94	94	94
NAA 10	ρ	−0.042	−0.113	−0.127	0.007
	*p*	0.691	0.282	0.226	0.945
	N	94	94	94	94
NAA 11	ρ	0.021	−0.051	−0.098	0.101
	*p*	0.843	0.628	0.350	0.337
	N	94	94	94	94
NAA 12	ρ	0.139	0.166	0.121	0.034
	*p*	0.183	0.111	0.249	0.745
	N	94	94	94	94

* *p* = 0.05

**Table 6:** Correlation of absolute concentrations of NAA obtained by long-echo MRS with biochemical indicators of metabolic syndrome in obese people.

Long-Echo		Triglycerides	HDL Cholesterol	Glucose	Insulin	HOMA-IR
NAA 1	ρ	−0.069	0.092	−0.075	−0.060	−0.083
	*p*	0.660	0.559	0.634	0.704	0.595
	N	44	44	44	44	44
NAA 2	ρ	0.209	0.056	−0.251	−0.068	−0.119
	*p*	0.178	0.723	0.105	0.667	0.449
	N	44	44	44	44	44
NAA 3	ρ	0.057	0.005	−0.059	−0.017	−0.046
	*p*	0.718	0.975	0.708	0.913	0.770
	N	44	44	44	44	44
NAA 4	ρ	−0.058	0.086	0.187	0.305 *	0.300
	*p*	0.713	0.582	0.230	0.047	0.051
	N	44	44	44	44	44
NAA 5	ρ	0.187	−0.177	0.289	0.118	0.210
	*p*	0.231	0.257	0.060	0.453	0.176
	N	44	44	44	44	44
NAA 6	ρ	−0.058	0.011	−0.057	−0.124	−0.111
	*p*	0.714	0.945	0.718	0.430	0.478
	N	44	44	44	44	44
NAA 7	ρ	0.014	0.091	−0.222	−0.410	−0.422 **
	*p*	0.930	0.560	0.152	0.006	0.005
	N	44	44	44	44	44
NAA 8	ρ	0.014	0.090	−0.222	−0.409 **	−0.422 **
	*p*	0.931	0.565	0.152	0.007	0.005
	N	44	44	44	44	44
NAA 9	ρ	0.079	0.070	0.184	0.027	0.091
	*p*	0.613	0.655	0.238	0.865	0.560
	N	44	44	44	44	44
NAA 10	ρ	0.007	−0.157	0.136	0.023	0.077
	*p*	0.963	0.313	0.386	0.882	0.621
	N	44	44	44	44	44
NAA 11	ρ	0.087	−0.084	0.040	−0.200	−0.136
	*p*	0.578	0.592	0.800	0.198	0.385
	N	44	44	44	44	44
NAA 12	ρ	−0.054	−0.004	−0.163	−0.006	−0.061
	*p*	0.732	0.981	0.297	0.970	0.696
	N	44	44	44	44	44

* *p* = 0.05; ** *p* = 0.01

**Table 7:** Correlation of absolute concentrations of Cho obtained by long-echo MRS with biochemical indicators of metabolic syndrome in obese people.

Long-Echo		Triglycerides	HDL Cholesterol	Glucose	Insulin	HOMA-IR
	ρ	−0.078	−0.104	0.126	−0.055	−0.012
Cho 1	*p*	0.619	0.506	0.420	0.726	0.938
	N	44	44	44	44	44
	ρ	0.020	0.134	−0.139	−0.018	−0.037
Cho 2	*p*	0.898	0.391	0.373	0.910	0.814
	N	44	44	44	44	44
	ρ	0.158	0.067	−0.091	−0.001	−0.035
Cho 3	*p*	0.310	0.667	0.562	0.993	0.823
	N	44	44	44	44	44
	ρ	−0.117	−0.096	0.156	0.246	0.269
Cho 4	*p*	0.455	0.539	0.318	0.111	0.081
	N	44	44	44	44	44
	ρ	0.320	−0.252	0.073	−0.008	0.033
Cho 5	*p*	0.036	0.102	0.643	0.958	0.836
	N	44	44	44	44	44
	ρ	0.222	−0.109	−0.065	−0.072	−0.042
Cho 6	*p*	0.152	0.487	0.678	0.647	0.791
	N	44	44	44	44	44
	ρ	0.133	0.060	−0.095	−0.392 **	−0.346 *
Cho 7	*p*	0.396	0.700	0.547	0.009	0.023
	N	44	44	44	44	44
	ρ	0.129	0.074	−0.110	−0.403 **	−0.357 *
Cho 8	*p*	0.411	0.638	0.483	0.007	0.019
	N	44	44	44	44	44
	ρ	0.112	−0.026	0.046	−0.244	−0.166
Cho 9	*p*	0.473	0.867	0.769	0.115	0.287
	N	44	44	44	44	44
	ρ	0.078	−0.099	−0.067	0.006	−0.015
Cho 10	*p*	0.617	0.527	0.668	0.970	0.923
	N	44	44	44	44	44
	ρ	0.227	−0.088	0.026	−0.185	−0.142
Cho 11	*p*	0.143	0.574	0.867	0.236	0.364
	N	44	44	44	44	44
	ρ	0.017	0.007	−0.268	−0.007	−0.073
Cho 12	*p*	0.916	0.966	0.082	0.964	0.640
	N	44	44	44	44	44

* *p* = 0.05; ** *p* = 0.01

## Data Availability

The human data supporting the findings of this study are not openly available due to privacy and sensitivity concerns. They are available from the corresponding author upon request.
